# True Mitotic Count Prediction in Gastrointestinal Stromal Tumors: Bayesian Network Model and PROMETheus (Preoperative Mitosis Estimator Tool) Application Development

**DOI:** 10.2196/50023

**Published:** 2024-10-22

**Authors:** Salvatore Lorenzo Renne, Manuela Cammelli, Ilaria Santori, Marta Tassan-Mangina, Laura Samà, Laura Ruspi, Federico Sicoli, Piergiuseppe Colombo, Luigi Maria Terracciano, Vittorio Quagliuolo, Ferdinando Carlo Maria Cananzi

**Affiliations:** 1 Department of Biomedical Sciences Humanitas University Pieve Emanuele, Milan Italy; 2 Pathology Department Istituto di Ricovero e Cura a Carattere Scientifico (IRCCS) Humanitas Research Hospital Rozzano, Milan Italy; 3 Sarcoma, Melanoma and Rare Tumors Surgery Unit Istituto di Ricovero e Cura a Carattere Scientifico (IRCCS) Humanitas Research Hospital Rozzano, Milan Italy

**Keywords:** GIST mitosis, risk classification, mHealth, mobile health, neoadjuvant therapy, patient stratification, Gastrointestinal Stroma, preoperative risk

## Abstract

**Background:**

Gastrointestinal stromal tumors (GISTs) present a complex clinical landscape, where precise preoperative risk assessment plays a pivotal role in guiding therapeutic decisions. Conventional methods for evaluating mitotic count, such as biopsy-based assessments, encounter challenges stemming from tumor heterogeneity and sampling biases, thereby underscoring the urgent need for innovative approaches to enhance prognostic accuracy.

**Objective:**

The primary objective of this study was to develop a robust and reliable computational tool, PROMETheus (Preoperative Mitosis Estimator Tool), aimed at refining patient stratification through the precise estimation of mitotic count in GISTs.

**Methods:**

Using advanced Bayesian network methodologies, we constructed a directed acyclic graph (DAG) integrating pertinent clinicopathological variables essential for accurate mitotic count prediction on the surgical specimen. Key parameters identified and incorporated into the model encompassed tumor size, location, mitotic count from biopsy specimens, surface area evaluated during biopsy, and tumor response to therapy, when applicable. Rigorous testing procedures, including prior predictive simulations, validation utilizing synthetic data sets were employed. Finally, the model was trained on a comprehensive cohort of real-world GIST cases (n=80), drawn from the repository of the Istituto di Ricovero e Cura a Carattere Scientifico (IRCCS) Humanitas Research Hospital, with a total of 160 cases analyzed.

**Results:**

Our computational model exhibited excellent diagnostic performance on synthetic data. Different model architecture were selected based on lower deviance and robust out-of-sample predictive capabilities. Posterior predictive checks (retrodiction) further corroborated the model’s accuracy. Subsequently, PROMETheus was developed. This is an intuitive tool that dynamically computes predicted mitotic count and risk assessment on surgical specimens based on tumor-specific attributes, including size, location, surface area, and biopsy-derived mitotic count, using posterior probabilities derived from the model.

**Conclusions:**

The deployment of PROMETheus represents a potential advancement in preoperative risk stratification for GISTs, offering clinicians a precise and reliable means to anticipate mitotic counts on surgical specimens and a solid base to stratify patients for clinical studies. By facilitating tailored therapeutic strategies, this innovative tool is poised to revolutionize clinical decision-making paradigms, ultimately translating into improved patient outcomes and enhanced prognostic precision in the management of GISTs.

## Introduction

Gastrointestinal stromal tumor (GIST) is the most common sarcoma type [1]. The majority harbors activating mutation in *KIT* or *PDGFRA* [2-5]. Even if these mutations represent early events in carcinogenesis—being shared by clinically irrelevant and very aggressive GIST [6,7]—they are the molecular basis for the very active tyrosine-kinase-inhibitor (TKI) therapy [8-10]. TKIs have revolutionized GIST treatment and have been used in all the disease stages since their introduction [11-14]. Notably, they can be administered as neoadjuvant treatment for patients with high-risk disease or for reducing the extent of surgery in GIST in a peculiar location (ie, rectum and duodenum) [14-17]. While neoadjuvant therapy with Imatinib is beneficial for patients with high-risk disease [18] and for those who may not achieve R0 resection or can undergo less mutilating, function-sparing surgery if there is a volumetric reduction [17], TKIs present certain challenges. One major issue is that they may impair accurate postoperative risk assessment, as tumor response to therapy can prevent correct risk evaluation [5]. Several risk assessments in GIST have been developed to identify size, site, and mitotic count as important features [19-21]. Indeed, mitotic count on the surgical specimen after TKI therapy can be greatly modified—especially in the case of tumor response. Therefore, in these patients, the subsequent management will be guided by a risk assessment computed with the mitotic count from the biopsy, and this can lead to mistreatment.

On the other hand, during initial patient management, the correct identification of high-risk patients can also fail: a very small amount of tissue on biopsy is required to make the GIST diagnosis since good immunohistochemical markers (rather specific and sensitive) exist [22-25]. Therefore, whereas size and site can be accurately assessed by imaging, mitotic count on biopsy can face several limitations, some purely biological (such as tumor heterogeneity) and others more physical (ie, the size of the specimen available for counting, which is a classic example of sampling bias) [26-30]. Thus, it may happen to incorrectly classify the risk of a GIST preoperatively, and this might lead to surprises after the mitotic count on the surgical specimen is performed, often due to an underestimation of the mitotic count [31-33].

These limitations underscore the critical need for innovative approaches to refine preoperative risk stratification in GIST, aiming to mitigate the risks of misclassification and subsequent therapeutic mismanagement. The discrepancies between preoperative risk assessments and postoperative findings underscore the imperative for precision tools that can dynamically estimate mitotic count on surgical specimens, enhancing the accuracy of patient stratification and treatment planning.

In line with this imperative, we aim to develop an advanced computational tool, termed PROMETheus (Preoperative Mitosis Estimator Tool), designed to predict mitotic count on surgical specimens. By leveraging state-of-the-art Bayesian modeling techniques and integrating comprehensive clinicopathological variables, PROMETheus seeks to address the limitations of current risk assessment methodologies, offering clinicians a reliable means to anticipate postoperative mitotic counts and refine preoperative treatment strategies effectively.

## Methods

### Modeling Strategy

#### Bayesian Network and Workflow

As a modeling strategy, we used the Bayesian network with the aim of predicting the mitotic count on the surgical specimen. Of note, we use the term “Bayesian network” to indicate the model’s graphical representation and the collection of functions necessary to use it for statistical learning. This loose definition is often used in practice; however, it is broader than the one defined by the term’s inventor, Judea Pearl. In his 2009 book *Causality*, he identifies the directed acyclic graphs (DAGs) with the term Bayesian networks: “Directed graphs, especially DAGs, have been used to represent causal or temporal relationships…and came to be known as Bayesian networks, a term coined…to emphasize three aspects: (1) the subjective nature of the input information; (2) the reliance on Bayes’ conditioning as the basis for updating information; and (3) the distinction between causal and evidential modes of reasoning [34]. In this paper, the meaning of “Bayesian network” is closer to the one of structural causal models (SCMs) [35]. Briefly, we designed an SCM of the variables, created a mock data set, wrote a probabilistic program, validated it on the data simulation, fit the model to the data, and compared multiple models with different structures; these procedures are often collectively referred as the Bayesian workflow [36-39].

#### Causal Modeling

The graphical representation of the Bayesian network was done with DAGs: the variables were represented by nodes and the conditional dependencies through directed edges. Based on the graphical representation, we built an SCM, which we used for data simulation and as the model for the fits.

#### Probabilistic Programming

We wrote and fit the models using R software (version 4.1.2; R Foundation for Statistical Computing) and Stan (version 2.21.0; Stan Development Team) [40-43]. Stan is a probabilistic programming language that runs a No U-Turn sampler, an extension to Hamiltonian Monte Carlo (HMC) sampling, which is itself a form of Markov Chain Monte Carlo (MCMC) [44]. To promote regularization and reduce overfitting, we used a multilevel-hierarchical modeling strategy [39,45,46]. Distributions (likelihood and priors) were chosen with maximum entropy criteria [39]. To understand priors’ implications, we run prior predictive simulations [37,38,46]. To minimize divergent transitions, we reparametrized the models with a noncentered equivalent form when appropriated [39,47]. To ensure a good representation of the sample space, we visually inspected the chains with trace plots and trankplots [48]. We then monitored the chains with postmodeling diagnostics such as the number of effective samples and the Gelman-Rubin convergence diagnostic 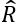.

[45,48]. Of note, 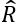. is defined in the cited references is different from the classic definition by Gelman and Rubin (1992). All models’ fits were plotted against the fitted data to ensure a good representation of the outcome space (posterior predictive check) [39,49,50]. Compatibility intervals (CIs) were calculated as the highest posterior density interval (HDPI) [51].

#### Model Selection

For the aim of pure prediction—as in our case—the best model can be selected based on information theory by estimating model performance using the widely applicable information criterion (WAIC, a generalization of Akaike information criteria) and Pareto Smooth Important Sampling Leave-One-Out Cross-Validation (PSIS-LOO-CV) criteria [52-54]. We checked that the 2 statistics gave the same results, leading us to trust their results [39,55,56]. We then selected the model with the lowest deviance in out-of-sample performance.

#### Forecasting

The posterior probability density of the coefficients was then used to create an application that, given the chosen variables, computed the posterior probability distribution in the outcome space (ie, the mitotic count on the specimen). Moreover, we programmed the application to calculate the risk class from this computed posterior distribution of the mitotic count.

### Study Population

#### Data Set

The cases came from a prospectively maintained database including all the patients who underwent surgery for primary sarcoma in the Istituto di Ricovero e Cura a Carattere Scientifico (IRCCS) Humanitas Research Hospital (Milan, Italy). This database comprises extended clinical and pathological information and contains 233 GISTs operated from January 2000 to March 2022.

### Inclusion and Exclusion Criteria

We included patients who had preoperative diagnostic biopsy and underwent surgical resection, with informed consent for research and available histologic material from both the biopsy and the surgical specimen. The histology was reviewed by a sarcoma pathologist (author SLR), and cases with diagnosis other than GIST were excluded.

### Pathology

#### Microscope Calibration

We calibrated the microscope with a stage micrometer slide and calculated the number of high-power fields (HPFs) needed to reach the size of 5 mm^2^. In line with the published guidelines, the number of HPFs to evaluate was 23.5 [30,57-59].

#### Mitosis

We defined mitosis as basophilic, dark, hairy material representing the chromosomes. Mitosis was counted when the chromosomes were either clotted (as at the beginning of metaphase), in a plane (as in metaphase and anaphase), or in separate clots (as in telophase), as previously described [60].

#### Biopsy Measurement

We measured the surface available under the microscope counting HPFs filled by neoplastic specimens up to 5 mm^2^ for very small biopsies we approximated the surface as a fraction of a field of view.

#### Tumor Response

Some of the series cases underwent preoperative therapy. To use these cases without polluting the estimate for mitotic count coefficient, we also recorded the response to treatment; this had a different meaning from a classical pathological response and was defined as follows: if a mitotically active area was identified on the surgical specimen (regardless of the size) and the mitotic count in this area was equal to or more than the biopsy count, the tumor was classified as “no response.” Therefore, this mitotic count on the biopsy was used in the model as if the case did not undergo preoperative therapy. Conversely, if the count on the surgical specimen was less than the count on the biopsy, the tumor was classified as “response” and the count was used to estimate a different coefficient that we did not use for prediction (see model description in the Results section and custom code for greater detail).

### Ethical Considerations

All patients signed an institutional written informed consent to research. The Independent Ethics Committee of IRCCS Humanitas Clinical Research reviewed the following project, giving approval on February 21, 2023 (145/23). Data were deidentified before analysis.

## Results

### Causal Modeling

**Figure 1:**
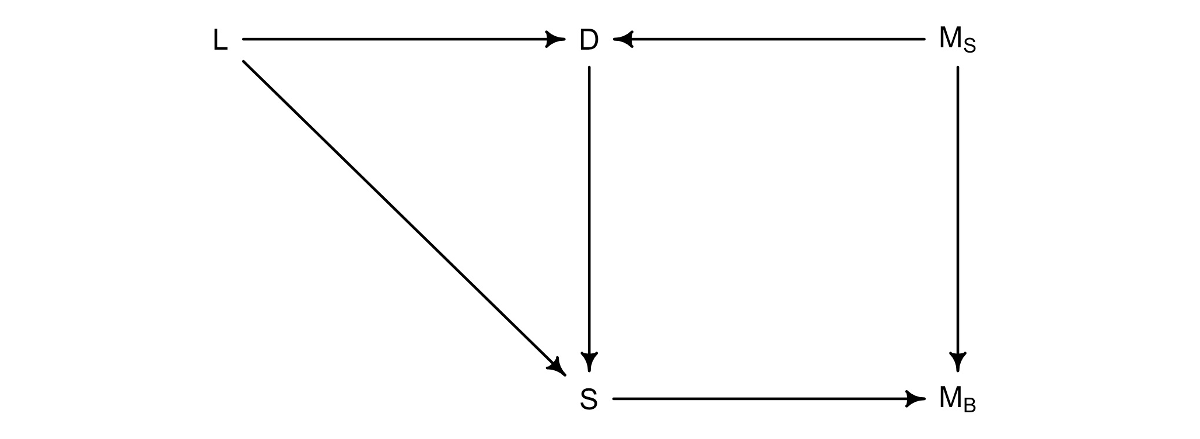
To determine the model covariates, we utilized a directed acyclic graph (DAG). In this causal framework, several factors influence the mitotic count on the surgical specimen. First, the dimension of the tumor impacts both the mitotic count on the biopsy and the surgical specimen. Larger tumors tend to exhibit higher mitotic activity. Second, the location of the tumor plays a crucial role in its growth pattern. For instance, gastric neoplasms often have more space to expand, leading to the development of symptoms with larger masses. Additionally, tumor location influences the accessibility of the biopsy site, as some sites are inherently more accessible than others. Finally, the amount of surface area available on the biopsy directly impacts the accuracy of the mitotic count estimation. A larger surface area allows for a more representative sampling of the tumor. This simplified causal model elucidates the relationship between various factors affecting GIST mitotic count. Please refer to the main text and supplementary materials for further details. In the model notation,*D*represents the dimension of the tumor, *L* denotes the location, *M*^B^ signifies the mitotic count on the biopsy, *M*^S^ indicates the mitotic count on the surgical specimen, and *S* represents the surface area of the biopsy.

We designed a causal model to identify the covariates to be included in the model for estimating the mitotic count on the surgical specimen. We assumed that the true mitotic count is the one counted on the surgical specimen (*M^S^*) The more tumor cell replicates, the bigger the tumor is (*D*). We also identified the anatomical location (*L*) as a cause of tumor dimension (*D*), in the sense that in certain locations the symptoms would appear earlier, thus influencing the measured size at diagnosis. Moreover, location directly causes the total amount of tissue available for evaluation at biopsy (ie, the measured biopsy surface, *S*); some sites are more difficult to reach than others. Finally, mitotic count on the biopsy (*M^B^*) reflects the mitotic count on the surgical specimen (*M^S^*) and due to tumor heterogeneity and sampling bias, the measurement on the biopsy also depends on the surface examined (*S*; Figure 1 and Multimedia Appendix 1 for an extended version).

### Probabilistic Modeling

Our inferential target was the mitotic count on the surgical specimen (*M^S^*); as the name suggests, it is a count variable; therefore, we chose a Poisson distribution to model it, as in Equation (1). Poisson distributions have just 1 parameter, *λ*. It is the expected value and the expected variance of the count variable, and it is the parameter used for the generalized linear model. It needs to be positive, and a common link function is to exponentiate the model. Given each patient *i*, we estimated a coefficient for the tumor dimension (*β*) and for surface of the biopsy (*γ*) for each location (*L*) using a multilevel-hierarchical model for both of them (*β_[L]_D_i_* and *γ_[L]_S_i_* respectively); the parameters *δ* and *
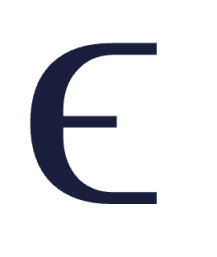
* were alternatively switched on and off by the presence of response to therapy *R*, as defined in the methods section; finally, we set an intercept *a*, as in Equation (2). To justify the prior choice, we used prior predictive simulation.



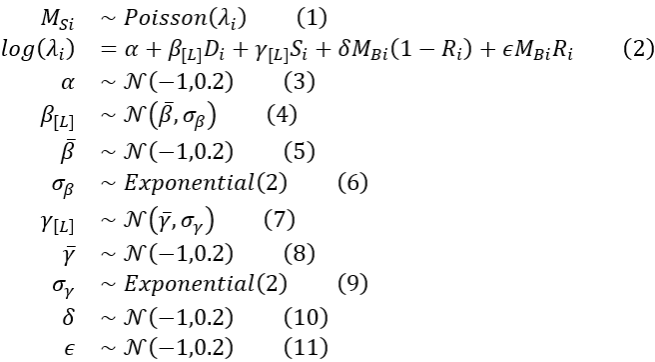



### Prior Predictive Simulation

**Figure 2:**
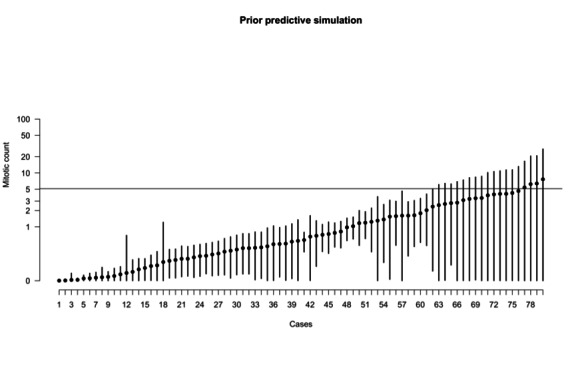
Prior predictive simulation of the λ parameter (ie, what the model anticipates before encountering the data). This plot displays 80 simulated cases derived from the priors. The majority of the expected values indicate a very low mitotic count, aligning with real-world expectations. However, the model is not startled by higher mitotic counts, even though it anticipates encountering them in a minority of cases without prior training.

The majority of GISTs are clinically irrelevant, with a mitotic count less than 5/5 mm^2^. A few of them can have a higher mitotic count, even to a greater order of magnitude; however, it is biologically implausible to expect many cases with a mitotic count greater than 50. Using this field-specific knowledge, we chose normal distributions for model coefficients, as seen in Equations (3), (5), (4), (7), (8), (10), and (11), and exponential distributions for scalar coefficients, as seen in Equations (6) and (9). Through serial simulations, we narrowed the numerical values. For a graphical representation of part of the coefficients, see Multimedia Appendix 2). The results of the prior predictive simulation show that most of the simulated *λ* have values that conform to the field-specific knowledge (Figure 2).

### Fitting the Mock Data Set

**Figure 3:**
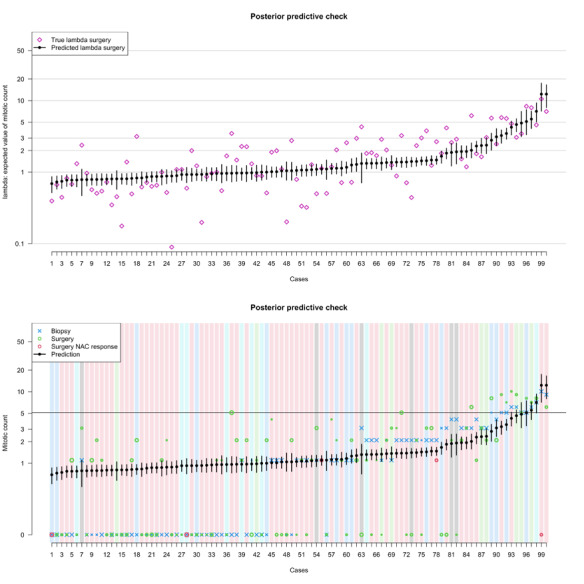
Posterior predictive simulation. The upper panel shows the imputed λ parameter for each sample against the ground truth; consider the logarithmic scale on the y-axis. The lower panel shows the same imputed λ parameter against the fitted data. On average, the mitotic count on either the surgical specimen or the biopsy is lower than the expected value. This is due to the asymmetry of the Poisson distribution. The size of the X is proportional to the biopsy surface, and the size of the empty dots is proportional to the tumor size. The color of the background corresponds to the tumor site. NAC: N-acetylcysteine.

To test the model performance, we fitted it on a simulated data set of 100 cases. The custom code to produce the mock data set is available on the cited repository. The fit with a centered parameterization resulted in 2% divergent transitions; therefore, we rewrote the model in a noncentered form. The fit diagnostics were satisfactory: 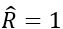. was obtained for all the parameters, and the energy from the Hamiltonian had a Gaussian outlook and the trankplots of the log-probability showed a satisfactory convergence of the chains (Multimedia Appendix 3). All the parameters had a satisfactory number of effective samples and a good outlook of the trankplots (Multimedia Appendix 4). The posterior probability density for each coefficient is depicted in Multimedia Appendix 5. To check the model fitness, we compared the inferred *λ* by the model to the true *λ* used for data simulation. This procedure revealed that the model regularized the values within each modeled site and was able to recover a value closer to *λ* even if provided with lower values (Figure 3).

### Fitting the Real Data Set

**Figure 4:**
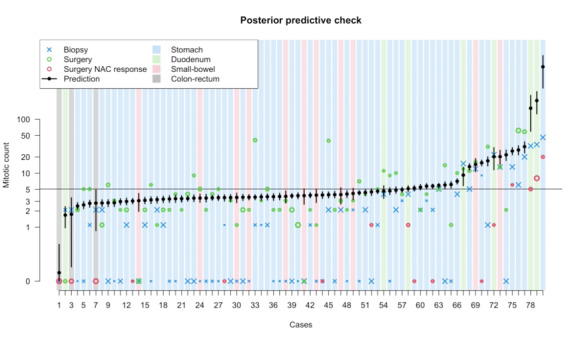
Posterior predictive simulation on the true data set. The size of the X is proportional to the biopsy surface, the size of the empty dots is proportional to the tumor size; the color of the background corresponds to the tumor site.

We then fitted the model to the real data. We were able to fit the data with the centered version of the model. Similarly to the simulated dataset, the fit’s diagnostics were satisfactory: 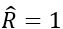. was obtained for all the parameters. The energy from the Hamiltonian had a Gaussian outlook and the trankplots of the log-probability showed a satisfactory convergence of the chains (Multimedia Appendix 6). Moreover, all the parameters had a satisfactory number of effective samples and a good outlook of the trankplots (Multimedia Appendix 7); the posterior probability density for the model coefficient is shown in Multimedia Appendix 8 and Table 1. The site (*L*) was an index variable with values from 1 to 4, representing coefficients for the colon-rectum, duodenum, small intestine, and stomach, respectively. Of note, the model posterior distribution for the biopsy count parameter (*δ*) is 1, consistent with our expectations. As in the data simulation, to see what the model learned, we moved to form the parameter space to the outcome space and simulated from the whole posterior distribution a *λ* parameter—that tells us the expected value of the Poisson distribution—for each case and plotted it against the data fitted. This posterior predictive simulation is depicted in Figure 4.

**Table 1:** Coefficients.

Parameter	Mean (SD)	5.5%	94.5%	N eff	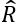
β_*L*[1]_	3.81 (1.91)	1.40	7.10	4396	1
β_*L*[2]_	0.12 (0.15)	–0.12	0.36	8407	1
β_*L*[3]_	0.30 (0.16)	0.03	0.55	5850	1
β_*L*[4]_	0.40 (0.04)	0.35	0.46	9032	1
γ_*L*[1]_	0.38 (0.44)	–0.25	1.13	6905	1
γ_*L*[2]_	–0.09 (0.15)	–0.33	0.15	8769	1
γ_*L*[3]_	–1.22 (0.34)	–1.77	–0.70	5884	1
γ_*L*[4]_	–0.17 (0.06)	–0.26	-0.08	9371	1
α	1.75 (0.05)	1.67	1.83	10178	1
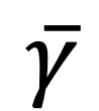	–0.85 (0.20)	–1.17	–0.53	8664	1
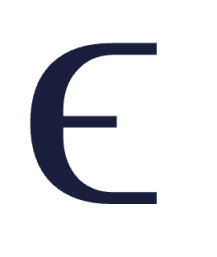	0.16 (0.05)	0.08	0.23	7978	1
δ	1.01 (0.08)	0.89	1.13	8031	1
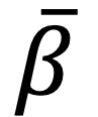	–0.90 (0.20)	–1.22	–0.57	9447	1
σ_γ_	0.89 (0.36)	0.44	1.55	6856	1
σ_β_	2.07 (0.74)	1.10	3.40	5274	1

### Model Selection

**Figure 5:**
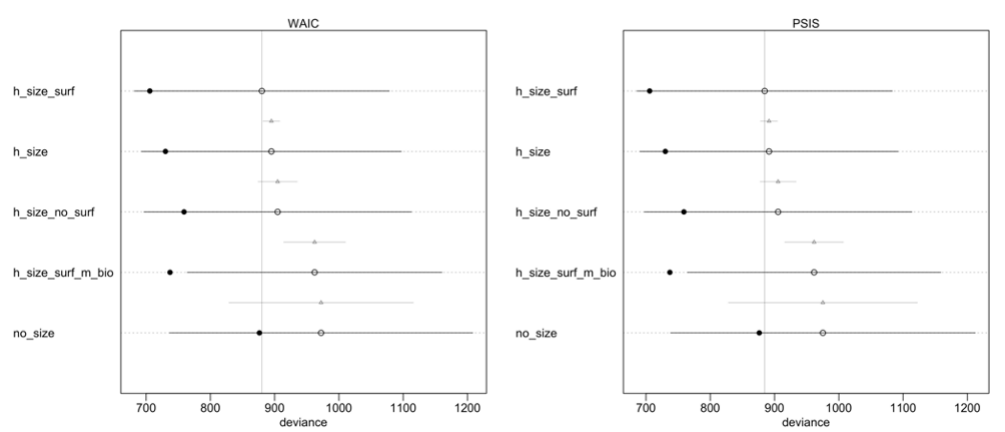
Model selection. The vertical line indicates the mean deviance of the reference model (the one with the lowest out of sample deviance). The filled dots are values within the sample. The empty dots represent the mean out-of-sample deviance with the bar indicating the 89% CI; the triangle is the contrast between the model and the reference model.

In general, adding parameters in the multilevel modeling reduces overfitting and improves the out-of-sample performance, whereas adding parameters without a hierarchical structure can reduce deviance within the sample, but results in lower out-of-sample performance (ie, increases overfitting). We therefore computed the deviance using WAIC and PSIS-LOO-CVC on our model (which had a multilevel hierarchical structure for the tumor size (*β*_L_) and biopsy surface (*γ*_L_) parameters. We also evaluated alternative models: one incorporating mitotic count on the biopsy with a hierarchical structure, found in Equation 12 in Multimedia Appendix 9; one with only tumor size with a hierarchical structure, as in Equation 13 in Multimedia Appendix 9; one with only tumor size without accounting for biopsy surface, as in Equation (14) in Multimedia Appendix 9; and one without a hierarchical structure without tumor size, as in Equation (15) in Multimedia Appendix 9. The model with a hierarchical structure for tumor size and biopsy surface parameters, as in Equation (2), had the lowest out-of-sample deviance (Figure 5). Therefore, we chose the model with a hierarchical structure for the tumor size and biopsy surface parameters to develop the application.

### Application Development

**Figure 6:**
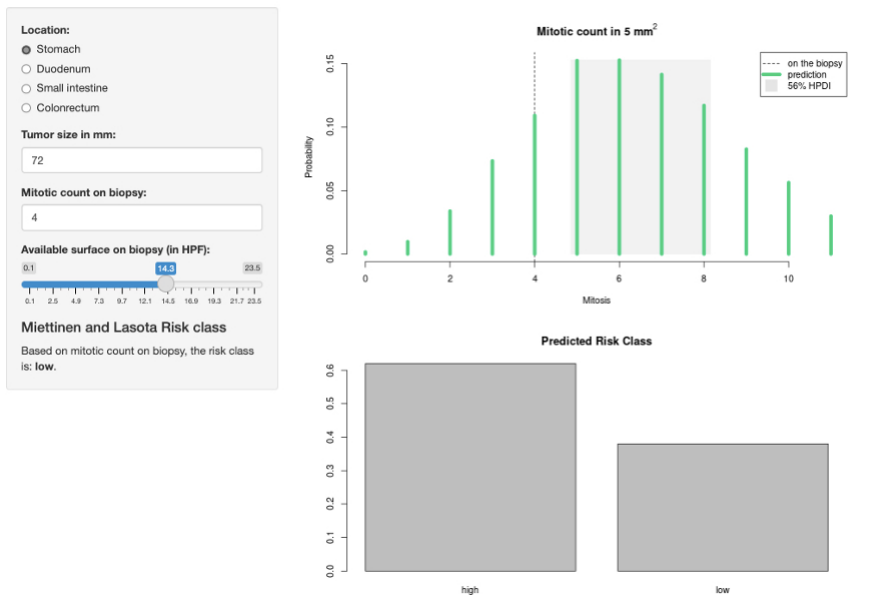
The PROMETheus (Preoperative Mitosis Estimator Tool) app. The image shows a screenshot for a gastrointestinal stromal tumor (GIST) measuring 72 mm with a biopsy surface of 14.3 high-power fields (HPFs; about 3 mm^2^). With these biological characteristics, the risk class is low. However, the model shows that the mitotic count is likely to be underestimated, with the most probable count on the surgical specimen predicted to be between 5 to 8 mitoses in 5 mm^2. Given this predicted mitotic count distribution, it is much more probable for the risk class to be high (more than 60%).

Using the posterior probabilities from the model, we developed PROMETheus, a web-based application [61]. The user interface has an input panel to insert the tumor location, tumor size, the mitotic count on the biopsy, and the available surface on the biopsy. Then, the application dynamically computes the risk class according to Miettinen and Lasota. Moreover, using the inputted data and the full posterior distribution, the application computes the expected mitotic count on the surgical specimen (indicating the most probable rendered counts) and the predicted risk class for the new posterior distribution of *M*^S^ provided (Figure 6).

### Development of the Preoperative Classification for GIST

To facilitate stage-adapted treatment planning and enhance data comparison across institutions, we developed a preoperative classification system for GIST based on the results of our computational model, PROMETheus. This classification system aims to provide a standardized definition of preoperative classification for GISTs, which can be used to guide therapeutic decisions and improve clinical trial designs (Table 2). The development of this classification included the probabilistic output of PROMETheus risk classification, important surgical parameters (such as site and size), and respectability.

**Table 2:** Proposed preoperative classification for GIST^a^.

Clinical type	GIST’s resectability	Features
0	Resectable	Gastric, mini tumor (<2 cm), PROMETheusb low risk
1	Resectable	Gastric, small tumor (2-10 cm), PROMETheus low risk
2	Resectable locally advanced	Large tumor (gastric: >10 cm; nongastric: >5 cm) Nongastric site Elevated risk of intraoperative rupture Need to mutilating or complex multivisceral resection PROMETheus high risk
3	Resectable metastatic	Resectable synchronous metastatic disease
4	Unresectable	Unresectable nonmetastatic disease

^a^GIST: gastrointestinal stromal tumor.

^b^PROMETheus: Preoperative Mitosis Estimator Tool.

## Discussion

### Principal Findings

Our study aimed to develop an innovative computational tool, PROMETheus, to accurately predict mitotic count on surgical specimens of GISTs, thereby addressing the challenges associated with preoperative risk assessment and treatment planning. The primary objective was to bridge the gap between clinical judgment and computed risk class, empowering clinicians to make informed decisions in complex scenarios. By using Bayesian networks and rigorous covariate selection methodologies, we succeeded in achieving this objective, providing clinicians with a novel approach to preoperative risk stratification in GISTs.

Our findings represent a potential advancement in the field of oncology, particularly in the context of sarcomas, where precise risk assessment is paramount for optimal treatment outcomes. By accurately predicting the mitotic count on surgical specimens, PROMETheus offers clinicians a tool to navigate the complexities of GIST management, enabling tailored treatment strategies based on individual patient characteristics. This approach aligns with the evolving paradigm of precision medicine, where treatment decisions are increasingly guided by molecular and pathological insights.

Mitotic count serves as a vital indicator of biological aggressiveness in oncology, playing a significant role in the grading systems of various tumors. With chemotherapy often administered to patients with high-grade tumors, mitotic count becomes a de facto predictive biomarker, providing valuable insights into treatment response. In the realm of GISTs, alongside size and site, the number of mitoses holds the utmost importance in current risk classifications [19-21]. However, unlike size and site, which can be easily assessed via imaging or endoscopy in the preoperative setting, accurately determining mitotic count poses challenges. Factors such as tumor heterogeneity and limited specimen size for counting (a classic example of sampling bias) hinder precise estimation during preoperative biopsy.

The introduction of effective therapies like Imatinib, which targets the molecular alterations driving GIST, has led to a clinical tendency to use this treatment preoperatively, even in cases where the risk of disease progression is not high. However, this approach has several drawbacks: (1) variable response: not all patients exhibit a decrease in tumor size, which means the extent of the surgery may remain unchanged; (2) side effects: Imatinib is not without side effects, highlighting the need for a tool to identify patients at genuine risk of metastasis who would benefit from neoadjuvant treatment; (3) risk classification after treatment: for patients treated with neoadjuvant Imatinib, it becomes challenging to perform risk classification on the surgical specimen, leaving uncertainty about the subsequent adjuvant therapy.

Integrating PROMETheus into clinical practice could have far-reaching implications for patient care. Not only does it enable more precise risk stratification in GISTs, but it could also facilitate stage-adapted treatment planning, ensuring that patients receive the most appropriate interventions based on their individual risk profiles. The preoperative classification system shown in Table 2 offers a common language for patient follow-up and treatment planning. It is a revision of previous work by our group [26], refined based on the insights gained from our study. This classification could lay the foundation for improved data collection and comparison across institutions, fostering collaboration and advancing research efforts in the field of GIST management.

### Comparison to Prior Work

Importantly, our study diverges from previous approaches that primarily focused on comparing biopsy and surgical specimens to evaluate the reliability of biopsy-based risk assessments. Instead, we recognized the inherent limitations of preoperative biopsy in predicting tumor grade, especially in the context of TKI therapy–induced tumor response. By focusing on predicting the mitotic count directly, our approach transcends these limitations, providing clinicians with a more accurate and reliable tool for preoperative risk assessment.

There are many tools for risk stratification in oncological practice, including in the sarcoma field, mainly based on nomograms, which are usually used to predict overall survival and the risk of metastasis [62]. While these tools have found utility in reanalyzing previous clinical studies and selecting patients for future trials [63], they are not tailored for GISTs. Our innovative approach stands out in 2 crucial ways. First, our Bayesian methodology empowers clinicians by providing the full posterior probability, capturing the inherent uncertainty often overlooked by traditional frequentist approaches that focus on central estimates. Second, unlike prognostic prediction tools, our method solely aims to forecast the mitotic count on the surgical specimen, a distinctive objective.

### Strengths and Limitations

Given the widespread use of mitotic count as a grading parameter, our approach holds potential for scalability across diverse clinical settings, such as breast cancer, solitary fibrous tumors, and soft tissue sarcoma [30,64]. The integration of our posterior probability with other tools could enhance their effectiveness. However, our choice of prediction methods was largely constrained by the data set’s size, as machine learning methods like deep learning demand substantial computational resources and larger sample sizes [65], which are often limited in rare diseases. Bayesian networks offer an advantageous alternative with principled variable selection, interpretability, and no minimum sample size requirement, making them a fitting choice for our study.

Despite our study’s promising findings, several limitations warrant consideration. First, our use of a complex multilevel hierarchical model, meticulously designed according to the DAG, introduces potential challenges in model efficacy and implementation. However, we addressed this complexity by subjecting the model to rigorous testing with simulated data, ensuring its robustness and reliability.

Second, the performance evaluation of PROMETheus is based on data from a relatively limited patient population, which may restrict the generalizability of our findings. However, unlike conventional approaches that rely solely on in-sample performance metrics for the model selection, we used PSIS-LOO-CV to identify the most effective model structure. Nonetheless, further validation on a larger and more diverse population is essential before considering the clinical deployment of PROMETheus, ensuring its efficacy and applicability across different clinical settings and patient demographics.

Third, the development and deployment of PROMETheus as a web-based application are at an early stage. Although we have made the tool publicly available as an open-source resource to encourage validation and application by other researchers and clinicians, the current version may lack certain functionalities and user-friendly features (such as the inclusion of risk classifications other than Miettinen and Lasota). Future work should focus on enhancing the application’s interface, usability, and integration with clinical workflows, as well as providing comprehensive user training and support to facilitate its adoption in clinical practice.

### Future Directions

We have developed a cutting-edge application that could revolutionize the estimation of mitotic count on surgical specimens, not only providing accurate quantification but also addressing the uncertainty that clinicians face when encountering challenging cases. By bridging the gap between clinical judgment and computed risk class based on available parameters, our application empowers clinicians to make informed decisions in complex scenarios. While previous studies have primarily focused on comparing biopsy and surgical specimens to evaluate the reliability of the former as the gold standard, we diverge from this approach. Our study transcends the limitations of preoperative biopsy in predicting tumor grade, shedding light on the underestimation of aggressiveness often associated with this method [66-69]. Embracing the widely accepted practice of causal modeling for covariate selection in epidemiological studies [70], we harness the power of Bayesian networks to pioneer a novel tool capable of predicting the mitotic count—a breakthrough in the existing literature.

In conclusion, our study represents a significant step forward in the development of precision tools for oncological risk assessment. By accurately predicting the mitotic count on surgical specimens in GISTs, PROMETheus enables clinicians to make informed treatment decisions, ultimately improving patient outcomes and advancing the field of sarcoma management. Moving forward, continued research and validation efforts will be essential to further refine and optimize the utility of PROMETheus in clinical practice, ultimately realizing its full potential in guiding personalized treatment strategies for patients with GISTs and other sarcomas.

## Appendix

Multimedia Appendix 1 A more elaborated directed acyclic graph (DAG). During optimization cycles of the modeling, we simplified both the DAG and the probabilistic program used. Indeed, our first modeling attempts included several latent variables (circled) as the true mitotic rate (*M*) and the biologic aggressiveness (U), and modeled in detail the disease presentation (including symptoms among the variables), the therapy indication and its response. Although this DAG is more accurate, its complexity impairs the parameter estimation efficiency. Moreover, within the domain of the Bayesian network, a different approach using a system of equations that translate the structural causal model (SCM) in the code for the fitting might have been used; however, the choice of a more simplified approach (ie, using the *M*^s^ as inferential target instead of as a variable) removes the need to refit the model to impute the value.

Multimedia Appendix 2 Prior predictive simulation for the model coefficients. The coefficients for tumor size and surface (β and γ, respectively) show a wider (t) distribution compared to the biopsy count (δ or  if a response to neoadjuvant therapy, N-acetylcysteine [NAC], is present). GIST: gastrointestinal stromal tumor.

Multimedia Appendix 3 Fit’s diagnostics on the simulated data set. The upper panel plots the number of effective samples against the R ^; the vertical gray line represents the total number of samples (half of the 8000 iterations: the first 4000 are discarded, multiplied for the 4 chains). The second panel shows the energy of the Hamiltonian, nicely following along a normal distribution (background blue line). The third panel shows a rank histogram plot (trankplot) for the log probability of the model. Each of the 4 chains alternates in dominating the ranking; this indicates that the chains mixed and converged.

Multimedia Appendix 4 Rank histogram plot (trankplot) for the all parameters. All the parameters have a nice mixing of the chains.

Multimedia Appendix 5 Model coefficients from fitting the simulated data set.

Multimedia Appendix 6 Fit’s diagnostics on the real gastrointestinal stromal tumor (GIST) cohort. The upper panel plots the number of effective samples against the R ^; the vertical gray line represents the total number of samples (half of the 4000 iterations multiplied for the 4 chains; the first 2000 are discarded). The second panel shows the energy of the Hamiltonian, nicely following along a normal distibution (background blue line). The trankplot for the log-probability of the model shows a nice mixing of the chains.

Multimedia Appendix 7 Rank histogram plot (trankplot) for all parameters. All parameters have a nice mixing of the chains.

Multimedia Appendix 8 Model coefficients from fitting the simulated data set.

Multimedia Appendix 9 Supplementary materials.

## Abbreviations

CI: compatibility interval
DAG: directed acyclic graph
GIST: gastrointestinal stromal tumor
HDPI: highest posterior density interval
HMC: Hamiltonian Monte Carlo
HPF: high-power field
IRCCS: Istituto di Ricovero e Cura a Carattere Scientifico
MCMC: Markov Chain Monte Carlo
PROMETheus: Preoperative Mitosis Estimator Tool
PSIS-LOO-CV: Pareto Smoothed Importance Sampling Leave-One-Out Cross-Validation Criteria
SCM: structural causal model
TKI: tyrosine-kinase inhibitor
WAIC: widely applicable information criterion

## Data Availability

Deidentified patient data are available upon motivated request on the public repository Zenodo [71]. Custom code for the analysis is available at the PROMETheus (Preoperative Mitosis Estimator Tool) GitHub repository [72].
