# Supplementary figures and images for: True Mitotic Count Prediction in Gastrointestinal Stromal Tumors: Bayesian Network Model and PROMETheus (Preoperative Mitosis Estimator Tool) Application Development
Source: J Med Internet Res. 2024 Oct 22;26:e50023. doi: 10.2196/50023 (PMC11538881; doi:10.2196/50023)

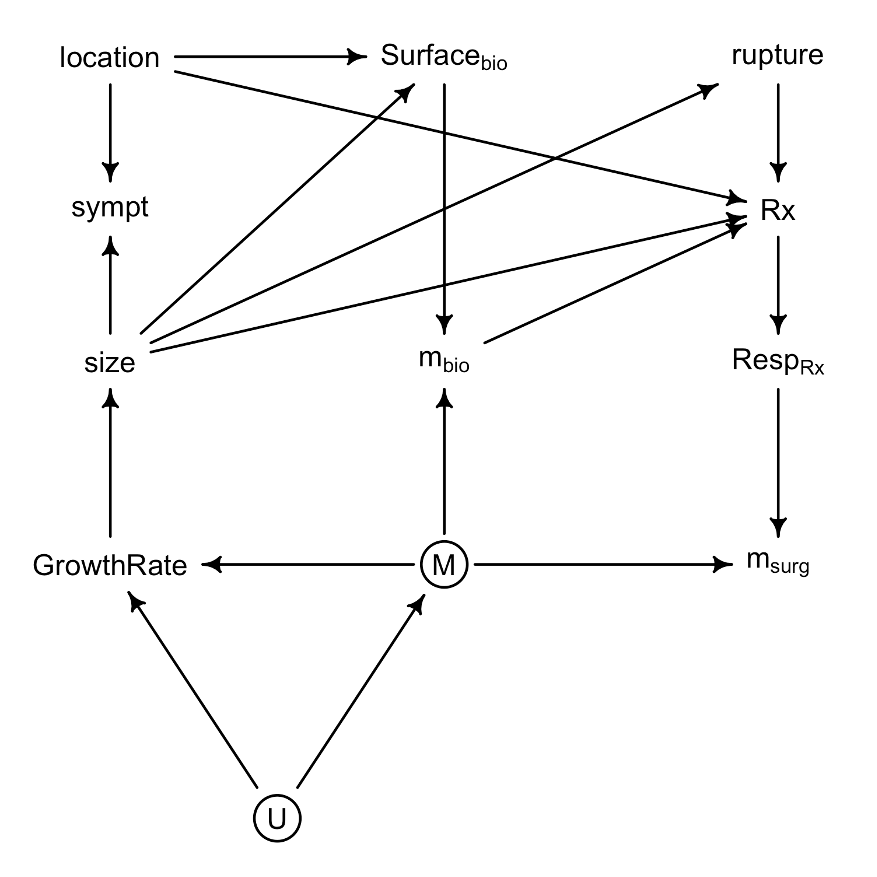

Supplement: Multimedia Appendix 1 [file jmir_v26i1e50023_app1.png]

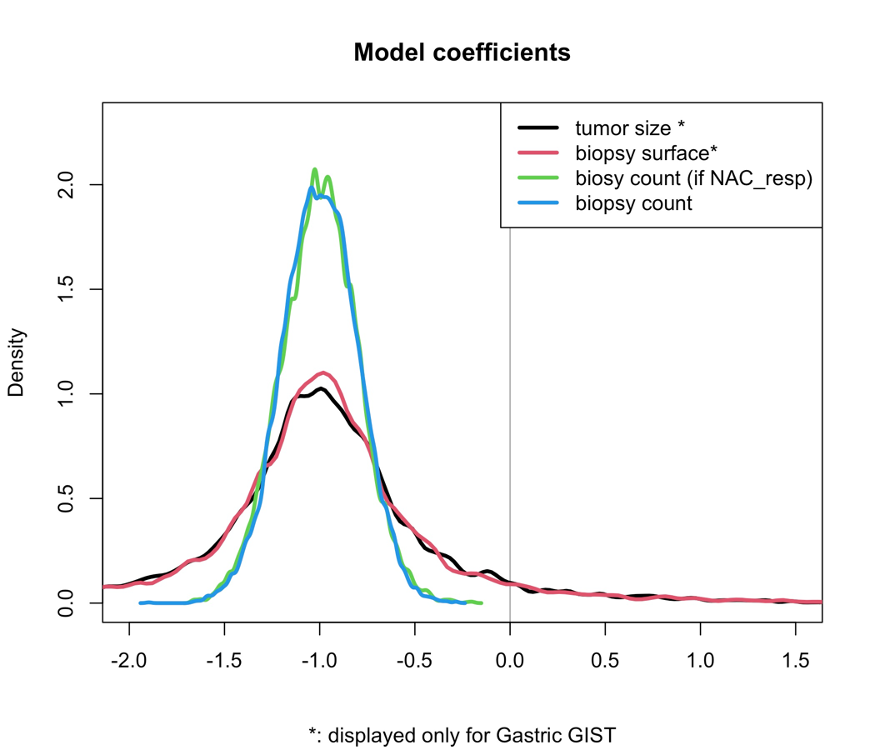

Supplement: Multimedia Appendix 2 [file jmir_v26i1e50023_app2.png]

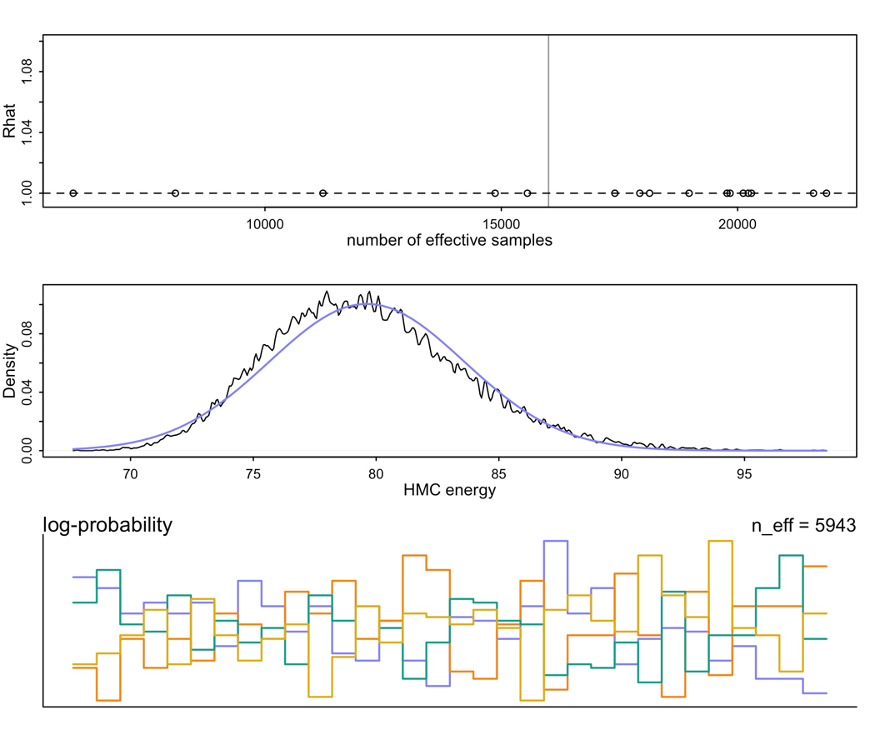

Supplement: Multimedia Appendix 3 [file jmir_v26i1e50023_app3.png]

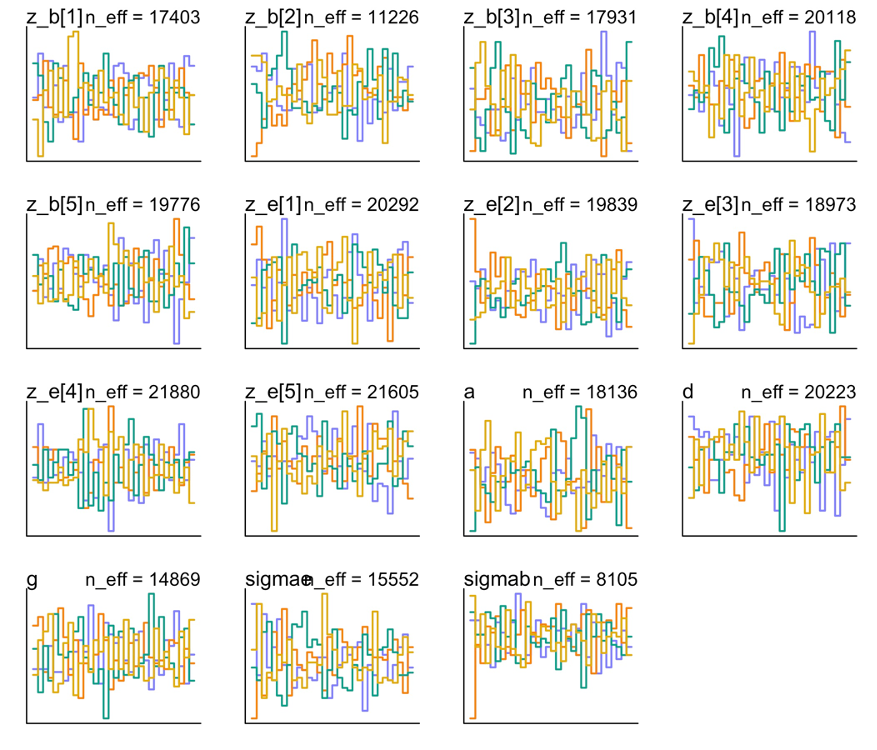

Supplement: Multimedia Appendix 4 [file jmir_v26i1e50023_app4.png]

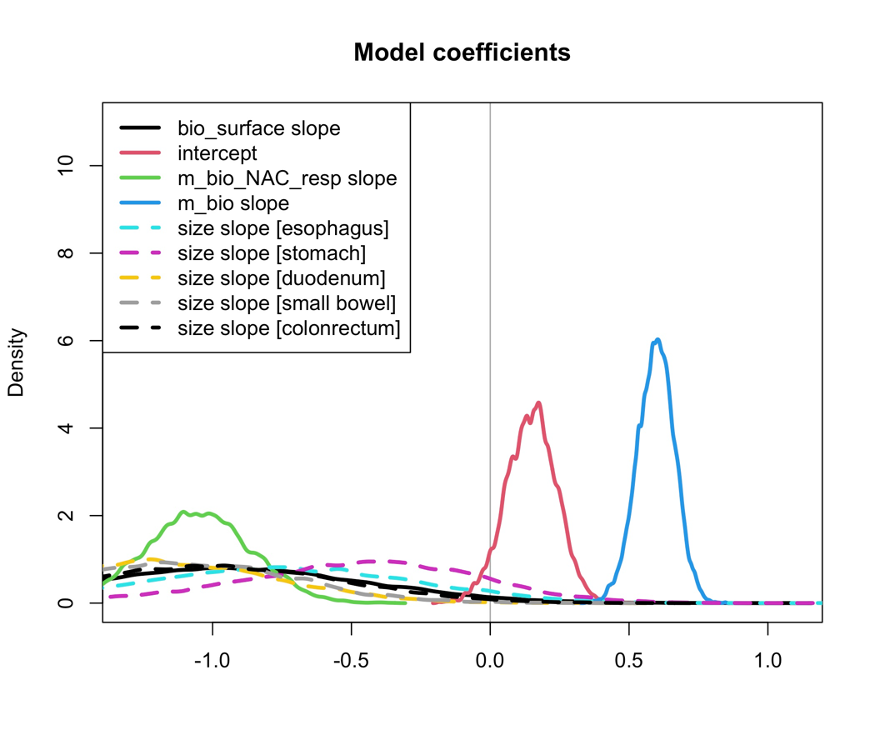

Supplement: Multimedia Appendix 5 [file jmir_v26i1e50023_app5.png]

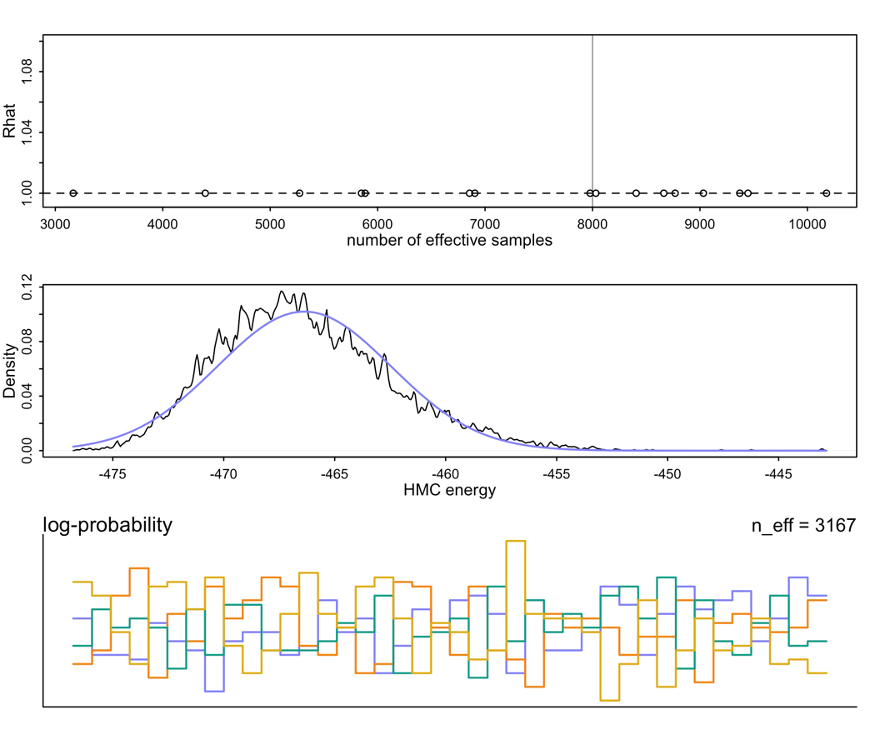

Supplement: Multimedia Appendix 6 [file jmir_v26i1e50023_app6.png]

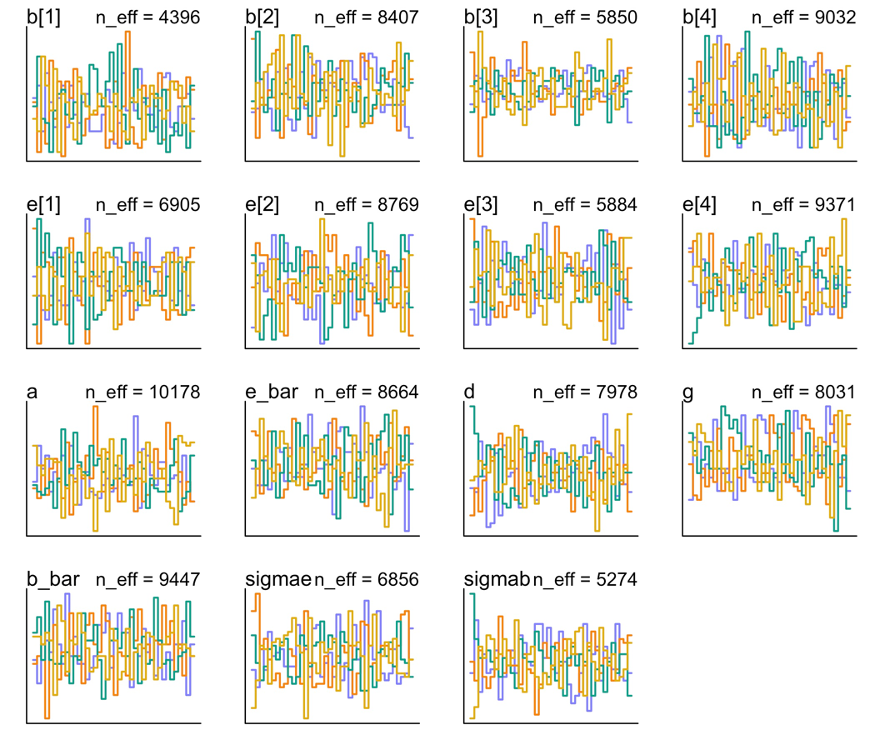

Supplement: Multimedia Appendix 7 [file jmir_v26i1e50023_app7.png]

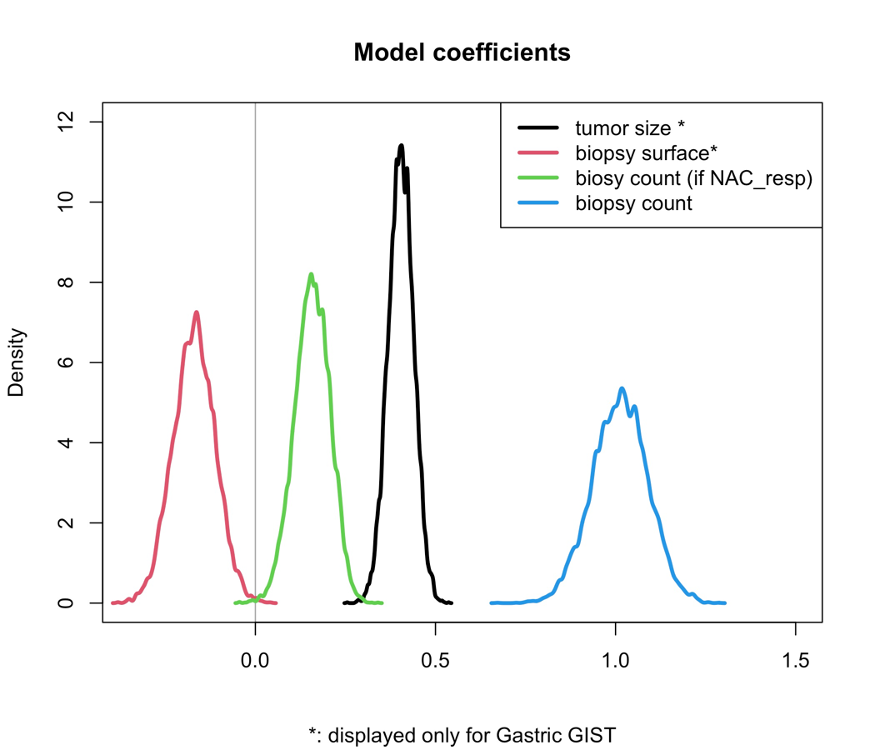

Supplement: Multimedia Appendix 8 [file jmir_v26i1e50023_app8.png]
