# Supplementary material for: True Mitotic Count Prediction in Gastrointestinal Stromal Tumors: Bayesian Network Model and PROMETheus (Preoperative Mitosis Estimator Tool) Application Development
Source: J Med Internet Res. 2024 Oct 22;26:e50023. doi: 10.2196/50023 (PMC11538881; doi:10.2196/50023)
Supplement: Multimedia Appendix 9 [file jmir_v26i1e50023_app9.docx]

## Alternative models

$$\begin{matrix} log\left( \lambda_{i} \right) & =\alpha+\beta_{\left[ L \right]}D_{i}+\gamma_{\left[ L \right]}S_{i}+\delta_{\left[ L \right]}M_{Bi}\left( 1-R_{i} \right)+\epsilon M_{Bi}R_{i} \left( 12 \right) \\ log\left( \lambda_{i} \right) & =\alpha+\beta_{\left[ L \right]}D_{i}+\gamma S_{i}+\delta M_{Bi}\left( 1-R_{i} \right)+\epsilon M_{Bi}R_{i} \left( 13 \right) \\ log\left( \lambda_{i} \right) & =\alpha+\beta_{\left[ L \right]}D_{i}+\delta M_{Bi}\left( 1-R_{i} \right)+\epsilon M_{Bi}R_{i} \left( 14 \right) \\ log\left( \lambda_{i} \right) & =\alpha+\gamma S_{i}+\delta M_{Bi}\left( 1-R_{i} \right)+\epsilon M_{Bi}R_{i} \left( 15 \right) \end{matrix}$$
